# Structural and functional characterization of a novel gene, *Hc-daf-22*, from the strongylid nematode *Haemonchus contortus*

**DOI:** 10.1186/s13071-016-1704-1

**Published:** 2016-07-29

**Authors:** Xiaolu Guo, Hongli Zhang, Xiuping Zheng, Qianjin Zhou, Yi Yang, Xueqiu Chen, Aifang Du

**Affiliations:** 1 College of Animal Sciences, Zhejiang Provincial Key Laboratory of Preventive Veterinary Medicine, Zhejiang University, Hangzhou, 310058 China; 2 Zhejiang Center of Animal Disease Control, Hangzhou, 310020 China; 3 Faculty of Life Science and Biotechnology, Ningbo University, Ningbo, 315211 China; 4 Present address: Institute of Preventive Veterinary Medicine, College of Animal Sciences, Zhejiang University, Hangzhou, Zhejiang 310058 China

**Keywords:** *Haemonchus contortus*, *Hc*-*daf*-*22*, *Caenorhabditis elegans*, β-oxidation, Diapause

## Abstract

**Background:**

The strongylid nematode *Haemonchus contortus* is a parasite of major concern for modern livestock husbandry because hostile environmental conditions may induce diapause in the early fourth-stage larvae.

**Methods:**

A new gene *Hc*-*daf*-*22* was identified which is the homologue of *Ce*-*daf*-*22* and human SCPx. Genome walking and RACE were performed to obtain the whole cDNA and genomic sequence of this gene. Using qRT-PCR with all developmental stages as templates to explore the transcription level and micro-injection was applied to confirm the promoter activity of the 5′-flanking region. Overexpression, rescue and RNA interference experiments were performed in N2, *daf*-*22* mutant (*ok 693*) strains of *C. elegans* to study the gene function of *Hc*-*daf*-*22*.

**Results:**

The full length gene of *Hc*-*daf*-*22* (6,939 bp) contained 16 exons separated by 15 introns, and encoded a cDNA of 1,602 bp (533 amino acids, estimated at about 59.3 kDa) with a peak in L3 and L4 in transcriptional level. The Hc-DAF-22 protein was consisted of a 3-oxoacyl-CoA thiolase domain and a SCP2 domain and evolutionarily conserved. The 1,548 bp fragment upstream of the 5′-flanking region was confirmed to have promoter activity compared with 5′-flanking region of *Ce*-*daf*-*22*. The rescue experiment by micro-injection of *daf*-*22* (*ok693*) mutant strain showed significant increase in body size and brood size in the rescued worms with significantly reduced or completely absent fat granules confirmed by Oil red O staining, indicating that *Hc*-*daf*-*22* could partially rescue the function of *Ce*-*daf*-*22*. Furthermore, RNAi with *Hc*-*daf*-*22* could partially silence the endogenous *Ce*-*daf*-*22* in N2 worms and mimic the phenotype of *daf*-*22* (*ok693*) mutants.

**Conclusion:**

The gene *Hc*-*daf*-*22* was isolated and its function identified using *C. elegans* as a model organism. Our results indicate that *Hc*-*daf*-*22* shared similar characteristics and function with *Ce*-*daf*-*22* and may play an important role in peroxisomal β-oxidation and the development in *H. contortus*.

## Background

The free-living nematode *Caenorhabditis elegans* normally develops from egg to adult through L1 to L4 larval stages in suitable environment. However, when worm density is too high or in starvation, L3 larva can transform into a dauer stage, in which worms would accumulate large amount of fat and have more spindly pharynx with thicker cuticle [1]. These physiological and metabolic changes can ensure a longer survival time for worms in harsh environments; normal worms can live for about two weeks while dauer worms can live for months [1]. In addition, these changes can be reversed when conditions become suitable for nematode growth, and worms can develop into regular reproductive adult stage [2]. *Caenorhabditis elegans* can sense population density through a group of small molecules named dauer pheromone which is secreted into environment [3]. The dauer pheromone consists of ascaroside derivatives with short, fatty-acid like side chains [3].

*Ce*-*daf*-*22* is homologous to the N-terminal portion of the invertebrate sterol carrier protein x (SCPx) [4], and UNC-24 is homologous to the C-terminal portion of the vertebrate SCPx [5] which is one of these lip/sterol intra cellular transport proteins [6] belonging to the well-characterized SCP2 gene family [7] and containing a sterol-binding domain (SCP2 domain). The vertebrate SCPx genes encode bipartite proteins containing 3-ketoacyl-CoA thiolase and sterol carrier protein-2 (SCP2) domains, located at the N- and C-terminus, respectively [8–10]. SCPx is found exclusively in peroxisomes [7]. It has been demonstrated that SCPx plays a unique role in the peroxisomal β-oxidation of branched-chain fatty acids [11] and in bile acid formation [4, 12]. In *C. elegans*, the *daf*-*22* mutant was originally identified as a mutant failed to induce dauer formation and was defective in peroxisomal β-oxidation and accumulated massive amounts of very-long-chain fatty acid (VLCFAs) and very long acyl-CoAs which are toxic [5]. The massive accumulation of fatty acids and their acyl-CoAs causes severe developmental defects. Researchers found that *Ce*-*daf*-*22* was also required for the biosynthesis of the short-chain fatty acid-derived side chains of the dauer pheromone [3], which indicated that there was certain link between dauer pheromone production and the general fatty acid metabolism [3]. Putative SCPx genes have been identified in parasitic nematodes *Ascaris suum* (ADY43045.1) and *Brugia malayi* [13] based on sequence similarities. However, the biological importance of SCPx in parasitic nematodes has not been yet reported.

*Haemonchus contortus* could also reach a special developmental stage in harsh environments such as low oxygen tension, short photoperiod, low temperature and host immune response, called diapause to avoid being expelled by hosts [2]. Little is known about details of the fatty acid β-oxidation pathway in this parasite and whether this pathway is also involved in the formation of diapause. Although significant progress in the molecular characterization of animal parasitic nematodes has been made during the last decade, the complexity of their life-cycles, the difficulties in collection of larvae of every stage, and the fact that adults cannot be cultivated in vitro, still make such studies difficult. *Caenorhabditis elegans* has been used as a heterologous expression system for defining the gene function of parasitic nematodes for many years. In this study, the complete cDNA of *Ce*-*daf*-*22* orthologue was first identified in *H. contortus*, with the name of *Hc*-*daf*-*22*. The 5′-flanking region of *Hc*-*daf*-*22* was examined for its promoter activity in *C. elegans* and the coding sequence containing the thiolase domain of *Hc*-*daf*-*22* was expressed in the *C. elegans daf*-*22* (*ok693*) strain by micro-injection in order to see whether it can rescue peroxisomal β-oxidation and accumulation of fat droplets in the intestine of *daf*-*22* loss-of-function mutant or not.

## Methods

### Strains

Adults of *H. contortus* (ZJ strain) were collected from sheep abomasa (sheep abomasa were obtained from the Hu Zhou Slaughter house), washed by phosphate buffer saline several times and stored in liquid nitrogen for later use. The *C. elegans* strains were maintained on NGM agar plates seeded with *E. coli* (OP50 strain) at 20 °C unless otherwise stated. The strains employed here were Bristol N2 and RB859 *daf*-*22 Y57A10C.6* (*ok693*) *II*. The *daf*-*22* (*ok693*) mutant used in this study was obtained from Caenorhabditis Genetics Center (Minneapolis, MN, USA). This mutant strain has a 790 bp deletion of *Ce*-*daf*-*22* gene and shows different ascaroside biosynthesis which leads to a phenotype of dauer pheromone production defect and fat associated bodies increase.

### Isolation of the full-length genomic cDNA of *Hc*-*daf*-*22*

The translated amino acids of *Ce*-*daf*-*22* were used to search the European Bioinformatics Institute’s parasite genomes database using tBLASTN algorithm. An EST of *H. contortus* (BF662749) with significant similarity to *Ce*-*daf*-*22* was identified. Full-length cDNA was obtained from total RNA drawn from *H. contortus* adults using 5′ and 3′ RACE kit (Takara, Dalian, China) according to the manufacturer’s instructions with gene-specific primers (Table 1). The products were cloned into pMD18-T vector and sequenced. To verify the full-length sequence of *Hc*-*daf*-*22*, primers were designed based on the results of 5′ and 3′ RACE to perform RT-PCR. The product was linked into pMD18-T vector and sequenced to confirm the whole sequence of this gene.

**Table 1:** Primers used in all experiments

Primer ID and direction	Primer sequence 5′–3′
*Hc*-*daf*-*22* 1F	TACAGACAGTGCCCGGCCATCA
*Hc*-*daf*-*22* 1R	GGCAGTAATAGGGGCAGGGAACA
*Hc*-*daf*-*22* 2F	GCTTCTGGCTCTTCTGGGCTCTACT
*Hc*-*daf*-*22* 2R	GAAAGCGGTAACTCTGACGTTGTGCTTGC
*Hc*-*daf*-*22* F1	ATGGGTAAATCAAAAGTATATG
*Hc*-*daf*-*22* R1	ACTGAGTGCAGATGGTT
*Hc*-*daf*-*22* F2	ACGCCGGCAAAGAACATAT
*Hc*-*daf*-*22* R2	CCCTTACCGACGTCACACACAAT
*Hc*-*daf*-*22* F3	ACTTATGAGGCTCTGGGATTG
*Hc*-*daf*-*22* R3	GCATCGATAGTCCACTTCTTTAC
*Hc*-*daf*-*22* F4	AGGAAAATGGGTGATCAATCCGTCT
*Hc*-*daf*-*22* R4	TCAGATTTTCGCTTTGAGCATTTTC
*Hc*-*daf*-*22*-SP1	GATAGGTATGCCAGTGAACCCGAGT
*Hc*-*daf*-*22*-SP2	GCAATCGTCCAACGCCATATTCACT
*Hc*-*daf*-*22*-SP3	AATACACTCACCGGCTTCCTTCACC
*Hc*-*daf*-*22* RT F	GTATATGTGATTGGTGTCGG
*Hc*-*daf*-*22* RT R	AATATCCGACGGTAGCTT
tub-F	TGTTCCATCACCCAAGGTATCC
tub-R	TGACAGACACAAGGTGGTTGAGAT
*Hc*-*daf*-*22*PF	AACTGCAGGATTGGTCGAGAGAGATGAAAC
*Hc*-*daf*-*22*PR	CGGGATCCGATGGTCCGGGCTGCAATAAATT
*Hc*-*daf*-*22d*F	TCCCCCGGGATGGGTAAATCAAAAGTATATG
*Hc*-*daf*-*22d*R	GGGGTACCCAGATTTTCGCTTTGAGCAT
*Ce*-PF	GCGCTCTAGATTAATTCAATGCACAACTC
*Ce*-PR	TCCCCCGGGTTTCTGGAACAATATT
RNAi F	CCCAAGCTTGTGAACAATGCCTGCCTGCGCTT
RNAi R	CTAGCTAGCCCTGTCGCACCGATGGG

### Acquisition of full-length genomic DNA of *Hc*-*daf*-*22*

Adult *H. contortus* worms, thawed from liquid nitrogen, were washed three times in phosphate buffer saline and homogenized in an ice bath. Total genomic DNA was extracted according to the protocol of Genomic DNA extraction kit (Takara, Dalian, China) with gene-specific primers (Table 1) designed based on the cDNA sequences and used to amplify the entire *Hc*-*daf*-*22* by long-PCR from total genomic DNA of adult *H. contortus* using LA Taq. The cycling conditions were: 94 °C for 1 min; 94 °C for 45 s; 60 °C for 30 s; 72 °C for 3 min for 30 cycles, with a final extension at 72 °C for 10 min. The PCR products were cloned into pMD18-T vector and assembled manually after carefully sequenced. The complete cDNA sequence and genomic DNA sequence were analyzed to determine intron/exon boundaries, following the AG-GT rule.

### Amplification of the 5′-flanking region of *Hc*-*daf*-*22* gene

The 5′-flanking region of *Hc*-*daf*-*22* was amplified by using a genome walking kit (Takara) with primers designed according to the genomic sequence acquired as described above. The primary PCR was carried out using an adapter primer AP1 and specific primer *Hc*-*daf*-*22*-SP1 with ~20 ng of total genomic DNA from *H. contortus* adults. The cycling conditions were: 1 min at 94 °C, 1 min at 98 °C, 30 s at 94 °C, 1 min at 65 °C, 2 min at 72 °C for 5 cycles; then 30 s at 94 °C, 3 min at 25 °C, 2 min at 72 °C, 30 s at 94 °C, 1 min at 65 °C, 2 min at 72 °C, 30 s at 94 °C, 1 min at 44 °C, 2 min at 72 °C for 15 cycles; followed by 10 min at 72 °C for the final extension. Primers AP1 and *Hc*-*daf*-*22*-SP2 were used at the second round of PCR using 1 μl first round PCR product as a template undergoing the following conditions: 15 cycles of 30 s at 94 °C, 1 min at 65 °C, 2 min at 72 °C, 30 s at 94 °C, 1 min at 44 °C, and 2 min at 72 °C; followed by 10 min at 72 °C for the final extension. Then 1 μl of the second round PCR product was used as template for the third round of PCR with primers AP1 and *Hc*-*daf*-*22*-SP3. The reaction conditions were the same as for the second round. PCR products of the third round were purified and cloned into pMD18-T vector for further sequencing.

### Sequence analysis and phylogenetic analysis

Homology of *Hc*-*daf*-*22* gene analysis was carried out using blastp at the National Center for Biotechnology Information [14]. Sequence similarities between the amino acid sequence of *Hc*-*daf*-*22* and other related species (*C. elegans*, *Brugia malayi* and *Ascaris suum*) were aligned using Clustal W method. Protein motifs were identified by searching the databases of PROSITE [14] and Pfam [15]. Phylogenetic analyses were performed using neighbor-joining (NJ) and maximum likelihood (ML) methods in Molecular Evolutionary Genetic Analysis (MEGA) [16].

### Determination of *Hc*-*daf*-*22* mRNA transcription levels in different developmental stages

Quantitative reverse transcription PCR (qRT-PCR) with specific primers was performed using different developmental stages of *H. contortus* as templates to determine the mRNA transcriptional levels (Table 1). Briefly, the total RNA of each sample, extracted by Trizol (Invitrogen, Shanghai, China), was used to synthesize the first-strand cDNA by random priming using ReverTra Ace-α (Cat. No. FSK-100, TOYOBO, Shanghai, China). The qRT-PCR (25 μl) using the THUNDERBIRD SYBR qPCR Mix (TOYOBO, Shanghai, China) in an ABI 7300 thermal cycler was performed under the following conditions: 50 °C for 2 min and 95 °C for 1 min for the first cycle followed by 95 °C for 15 s, 60 °C for 15 s and 72 °C for 31 s for 40 cycles. The dissociation curve was generated under the following conditions: 95 °C for 15 s, 60 °C for 1 min, 95 °C for 15 s and 60 °C for 15 s. Each sample was applied in triplicate using β-tubulin 8–9 (Accession: M76493) gene as a normaliser using specific primers (Table 1). The whole experiment was repeated three times. The mean threshold cycle (CT) values were used for further analysis.

### *Hc*-*daf*-*22* promoter amplification and transformation of *C. elegans*

The affliction of *Hc*-*daf*-*22* gene promoter was carried out using specific primers (Table 1) which were designed based on the 5′-flanking region of *Hc*-*daf*-*22*. The PCR product was purified and cloned into the upstream of *gfp* region of the pPD 95.77 expression vector to verify the ability of driving the green fluorescent protein (GFP) expression in *C. elegans*. This recombinant plasmid, designated as pPD 95.77*Hc*-*daf*-*22*-prom, was microinjected into the gonad of young adults of the wild type (N2) hermaphrodites as described together with another plasmid pRF4, which bears a dominant mutant allele of the *rol*-*6* gene, each at a final concentration of 50 μg/ml. The progeny worms showing the roller phenotype were collected to determine the expression patterns of GFP using a compound fluorescence microscope (Olympus IX71).

### Expression of *Hc*-*daf*-*22* in N2 and *daf*-*22* (*ok693*) strains of *C. elegans*

The coding sequence containing the thiolase domain of *Hc*-*daf*-*22* was amplified by PCR with primers listed in Table 1 with *Sma*I/*Kpn*I sites underlined. The product was purified and cloned into pPD 95.77 vector between the 2061 bp *Ce*-*daf*-*22* promoter region and the *gfp* region. The promoter region was amplified by PCR from N2 strain genomic DNA using the primers listed in Table 1 with restriction sites *Xba*I and *Sma*I underlined. The recombinant plasmid was microinjected into N2 and *daf*-*22* (*ok693*) strains as described above. Transgenic worms displaying the roller phenotype were further analyzed for examination of GFP activity as described above. *Ce*P-pPD95.77-*Hc*-*daf*-*22d* (pPD95.77 vector with *Ce*-*daf*-*22* gene promoter region and *Hc*-*daf*-*22* gene functional domain region) was also used as a rescue plasmid, which was microinjected into *daf*-*22* (*ok693*) mutants as described above. Transformants were selected based on GFP expression and rescued homozygous mutants were identified by fat staining, body size [17], growth rate [18] and brood size [19] measurements.

### Fat staining

Oil red O staining was performed as described [20]. Briefly, 200–300 young adult worms were washed three times with 1× PBS and settled by gravity. Worms were resuspended with 120 μl of PBS, together with an equal volume of 2× MRWB buffer containing 2 % paraformaldehyde (PFA) to permeabilize the cuticle. The 2× MRWB buffer contained the following: 160 mM KCl, 40 mM NaCl, 14 mM Na_2_EGTA, 1 mM spermidine-HCl, 0.4 mM spermine, 30 mM Na-PIPES pH 7.4, 0.2 % β-mercaptoethanol. Worms were gently rocked for 1 h at room temperature, and allowed to settle by gravity. Worms were then washed with 1× PBS to remove PFA one time, and resuspended in 60 % isopropanol for 15 min at room temperature to dehydrate. Isopropanol was removed after the worms had settled and worms were incubated overnight with rocking after adding 1 ml of Oil red O staining solution. On the following day, the staining solution was removed and 200 μl of 1 × PBS 0.01 % Triton X-100 added. Worms were photographed with an Olympus color camera equipped with DIC optics.

### *Hc*-*daf*-*22d* RNA interference

*Hc*-*daf*-*22d* was 857 bp, from 237–1,093 bp which contained a functional thiolase domain of *Hc*-*daf*-*22* gene. This fragment was amplified with specific primers (Table 1) containing the restriction sites *Hind* III and *Nhe*. The product was purified and cloned into the L4440 vector. The recombinant plasmid was transformed into HT115 (DE3) cells, an RNase III-deficient *Escherichia coli* strain with isopropyl-β-D-thiogalac-topyranoside-inducible T7 polymerase activity. The empty L4440 vector was also transformed into HT115 cells as control. Experiments were carried out using the standard bacterial feeding protocol [21] and repeated at least three times. Worms were observed under a microscope and any change in fat storage was confirmed by Oil red O staining.

### Statistical analyses

Statistical analysis for *Hc*-*daf*-*22* mRNA transcription levels and parameters of *C. elegans* were carried out using one-way ANOVA in SPSS 16.0. *P*-values < 0.05 were considered statistically significant. Graphs were made by GraghPad Prism 6.

## Results

### Characterization of *Hc*-*daf*-*22* gene from *H. contortus*

**Fig. 1:**
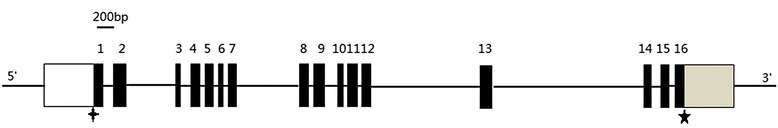
Genomic structure of *Hc*-*daf*-*22*. The numbered boxes are exons; blank box is 5′-UTR; grey box is 3′-UTR. The four-point star indicates translation start codon ATG, and the five point star represents translation stop codon TAA

**Fig. 2:**
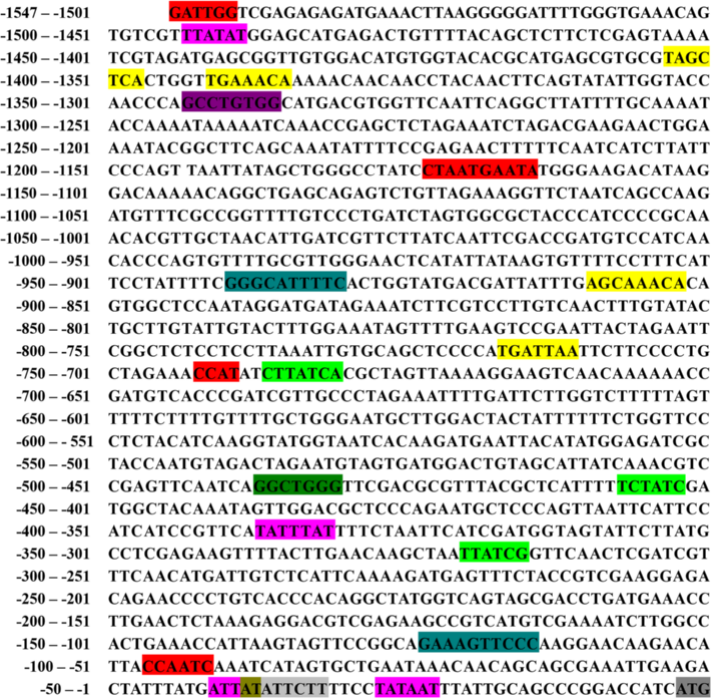
Upstream domain of the *Hc*-*daf*-*22* gene. The 5′-flanking region of *Hc*-*daf*-*22* gene was obtained and analyzed for putative transcription factor binding sites using the Transcription Element Search System (TESS). The main predicted promoter elements are highlighted in different colors: TATA box (*pink*); GATA-1 binding sites (*bright green*); CAC-binding protein sites (*green*); CCAAT binding factor (*red*); Unc-86 (*light grey*); AP-1 (*yellow*); AP-2 (*violet*); NFγB (*teal*). The start codon ATG is highlighted in *dark grey*

**Fig. 3:**
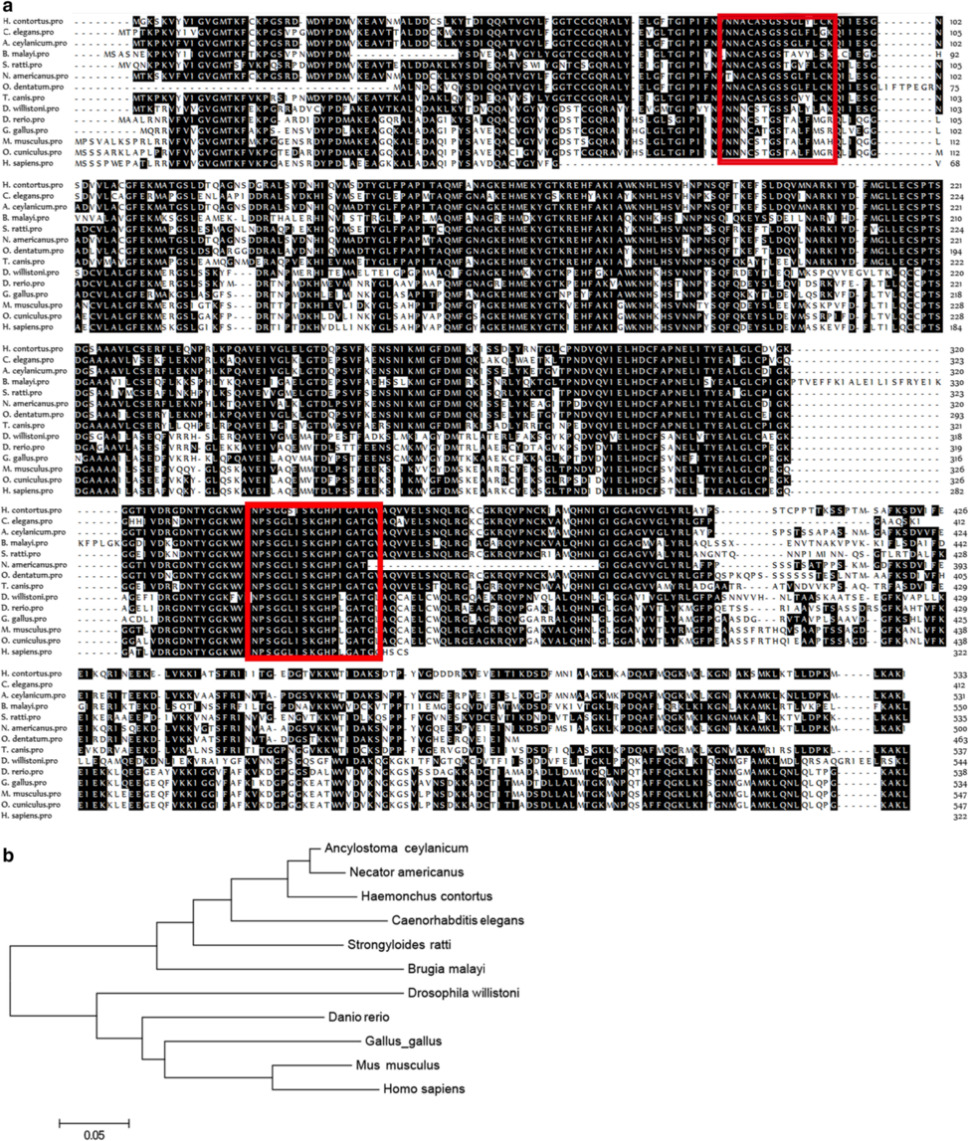
Characterization of *Hc*-*daf*-*22* gene from *H. contortus*. **a** Alignment of the deduced amino-acid sequences of Hc-DAF-22 and other species. Protein sequences were aligned using CLUSTAL W and BoxShade. Shading in black indicates residues that are consistent. Red boxes represent thiolase acyl-enzyme intermediate signatures, the first box represents the thiolases aryl-enzyme intermediate signature and the second box represents the thiolase-2 thiolase signature. The accession numbers representing these sequences are: NP 496639.1 (*C. elegans*), EYC23922.1 (*A. ceylanicum*: *Ancylostoma ceylanicum*), XP 001902124.1 (*B. malayi*: *Brugia malayi*), CEF60652.1 (*S. ratti*: *Strongyloides ratti*), XP_013305621.1 (*N. americanus*: *Necator americanus*), KHJ80319.1 (*O. dentatum*: *Oesophagostomum dentatum*), KHN79232.1 (*T. canis*: *Toxocara canis*), XP_002065586.1 (*D. willistoni*: *Drosophila willistoni*), NP_957159.1 (*D. rerio*: *Danio rerio*), NP_001292129.1 (*G. gallus*: *Gallus gallus*), NP_035457.1 (*M. musculus*: *Mus musculus*), NP_001075504.1 (*O. cuniculus*: *Oryctolagus cuniculus*), NP_001007099.1 (*H. sapiens*: *Homo sapiens*). **b** Phylogenetic tree based on deduced amino acid sequences of Hc-DAF-22 and its homologues from other species. NP 496639.1, AAB06496.1 (*C. elegans*), EYC23922.1 (*A. ceylanicum*: *Ancylostoma ceylanicum*), XP 001902124.1 (*B. malayi*: *Brugia malayi*), CEF60652.1 (*S. ratti*: *Strongyloides ratti*), XP_013305621.1 (*N. americanus*: *Necator americanus*), XP_002065586.1 (*D. willistoni*: *Drosophila willistoni*), NP_957159.1 (*D. rerio*: *Danio rerio*), NP_001292129.1 (*G. gallus*: *Gallus gallus*), NP_035457.1 (*M. musculus*: *Mus musculus*), NP_001007099.1 (*H. sapiens*: *Homo sapiens*)

A 1,777 bp transcript was obtained by overlapping 3′ and 5′ RACE results, including a 1,602 bp open reading frame (GenBank: HQ738470.1), a 58 bp 5′UTR and a 117 bp 3′ UTR. The whole gene (from the ATG initiation codon to the TAA stop codon) was 6,939 bp and consisted of 16 exons separated by 15 introns (Fig. 1). The 1,548 bp 5′-flanking region of *Hc*-*daf*-*22* was obtained by genome walking. Further analysis indicated that the sequence contained a TATA box, GATA-1, CCAAT binding factor, Unc-86, AP-1, AP-2, and NFγB sites (Fig. 2). The *Hc*-*daf*-*22* ORF encoded a 533-amino-acid protein (GenBank: AEO14647.1) with a mass of 59.2 kDa as predicted in the PROSITE database, which included the thiolases acyl-enzyme intermediate signature and thiolases signature 2 (Fig. 3a). The sequence was aligned with the DAF-22 protein of *C. elegans*, the sterol carrier protein of *Brugia malayi*, the non-specific lipid-transfer protein of *Ascaris suum*, sterol carrier proteins of chicken, *Drosophila melanogaster*, *Homo sapiens* and *Mus musculus* which showed similarities as follows: 80.3 %, 61.9 %, 73.0 %, 47.5 %, 45.6 %, 47.8 % and 48.2 %, respectively (Fig. 3a). Hc-DAF-22 showed a significantly higher similarity to parasitic nematodes and the Ce-DAF-22 in amino acids (Fig. 3b).

### Transcriptional level of *Hc*-*daf*-*22* throughout the lifespan of *H. contortus*

**Fig. 4:**
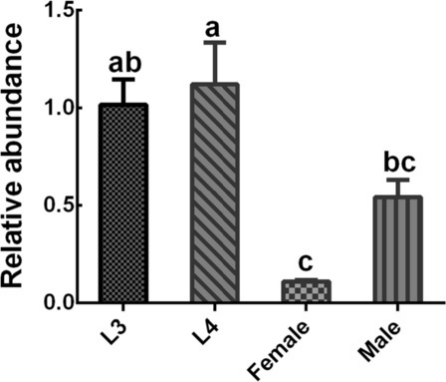
Transcriptional profile of *Hc*-*daf*-*22* at different developmental stages of *H. contortus* and genders. Data shown are mean values (± standard error of the mean, SEM) derived from three replicates in repeat experiments. All gene expression levels were normalized to those of the β-tublin gene. *P*-values are calculated by Dunnett’s test. There were significant differences between stages indicated by different letters (**a**, **b**, **c**) (*P* < 0.05)

The transcription level of *Hc*-*daf*-*22* was determined at different stages throughout the lifespan of *H. contortus* by qRT-PCR. *Hc*-*daf*-*22* was transcribed at detectable levels at L3, L4, adult male and female stages, with a peak in L3 and L4 stages. There was no significant difference in expression levels between male and female adults (t-test: *t* = 0.2052, *df* = 4, *P* = 0.8474) (Fig. 4).

### Promoter activity analysis of the 5′-flanking region of *Hc*-*daf*-*22* in *C. elegans*

**Fig. 5:**
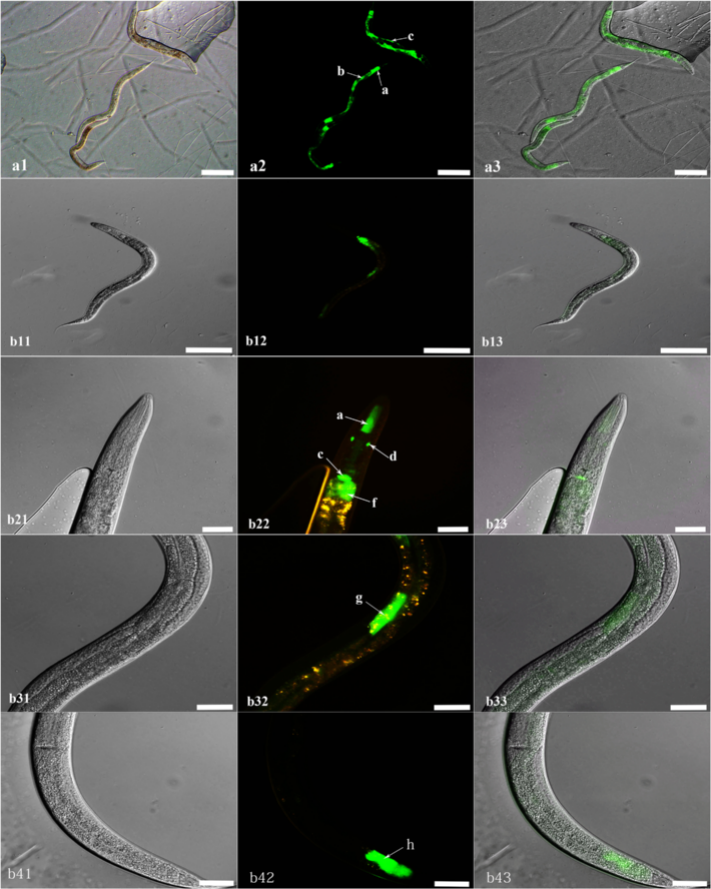
Expression pattern of *Ce*-*daf*-*22* and *Hc*-*daf*-*22* promoter GFP fusion in *C. elegans*. **a1**-**a3** Total view of *Ce*-*daf*-*22* promoter-derived worms showing pharynx (*arrow a*), intestine (*arrow b*) and hypodermis (*arrow c*) enrichment of GFP; **b11**-**b13** Total view of *Hc*-*daf*-*22* promoter- derived worm, **b21**-**b43** Views at higher magnification: *Hc*-*daf*-*22*P::GFP protein localized mainly in the pharynx (*arrow a*), excretory cell (*arrow d*) and intestine (*arrows e*, *f*, *g*, *h*). *Scale*-*bars*: 200 μm

The N2 strain of *C. elegans* was transformed by microinjection with the reconstructed plasmid as described above. The *Ce*-*daf*-*22* promoter was microinjected as control. Transgenic lines exhibiting the roller phenotype were selected. Fluorescence microscopy showed that GFP was mainly localized in the distal, middle and anterior part of the intestine, pharynx and excretory cells (Fig. 5) in the transgenic *Hc*-*daf*-*22*-*promoter*::*gfp* worms. However, compared to transgenetic *Ce*-*daf*-*22*-*promoter*::*gfp* worms, which could drive GFP expression in the hypodermis and throughout the intestine (Fig. 5), the level of GFP expression was clearly lower as we can see in the picture.

### Expression of *Hc*-*daf*-*22* in N2 strains

**Fig. 6:**
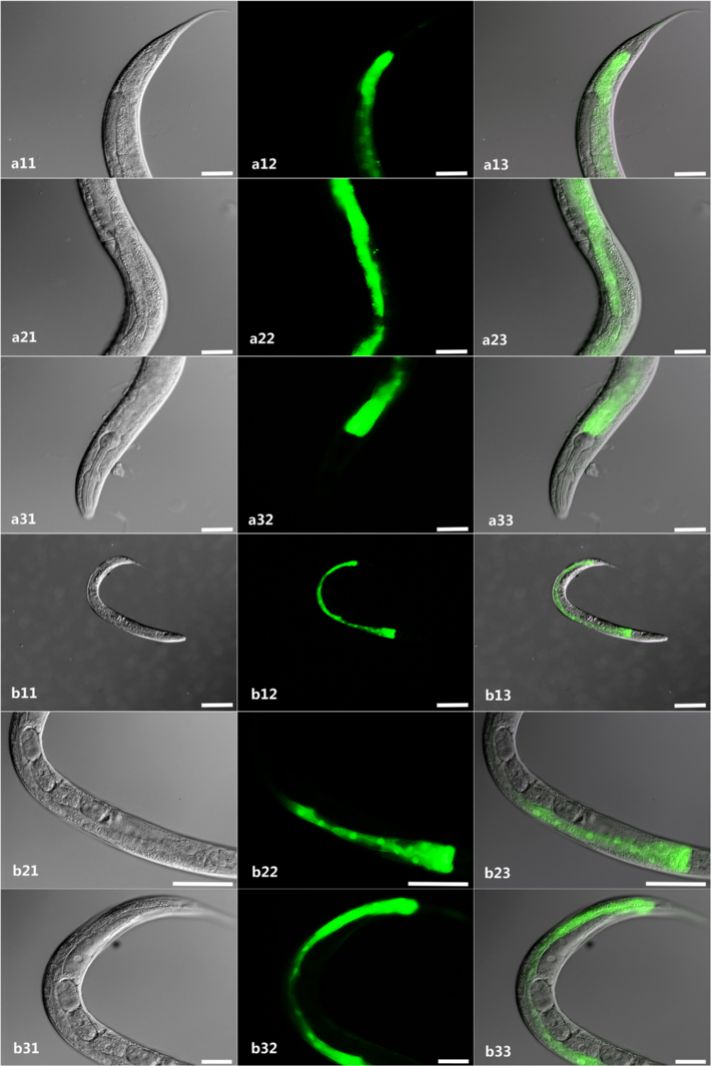
Hc-DAF-22 is located in intestinal cells in *C. elegans* N2 worms using *Ce*-*daf*-*22* promoter. The plasmid vector CeP-pPD95.77-*Hc*-*daf*-*22* which contained the 2,061 bp region upstream of the *Ce*-*daf*-*22* initiation codon and 1,105 bp region of the *Hc*-*daf*-*22* coding sequence was constructed and injected into wild type N2 worms. The expression pattern of Hc-DAF-22 in the N2 strain is shown, GFP could be seen throughout the whole intestine in both larvae and adults with high intensity. **a11**-**a33** L4 larvae; **b11**-**b33** adult worms. *Scale*-*bars*: 200 μm

In order to investigate the gene function of *Hc*-*daf*-*22* in vivo, the *Hc*-*daf*-*22* coding region was overexpressed in N2 transgenetic line, using *Ce*-*daf*-*22* 5′-flanking region as promoter. As the results showed, the expression of *Hc*-*daf*-*22*::*gfp* fusion protein could be observed in all life-cycle stages (L1, L2, L3, L4 and adult). GFP was mainly localized in the whole intestine, and more strongly, in the intestinal nuclei (Fig. 6).

### Rescue experiment of *daf*-*22* (*ok693*) strain with *Hc*-*daf*-*22*

**Fig. 7:**
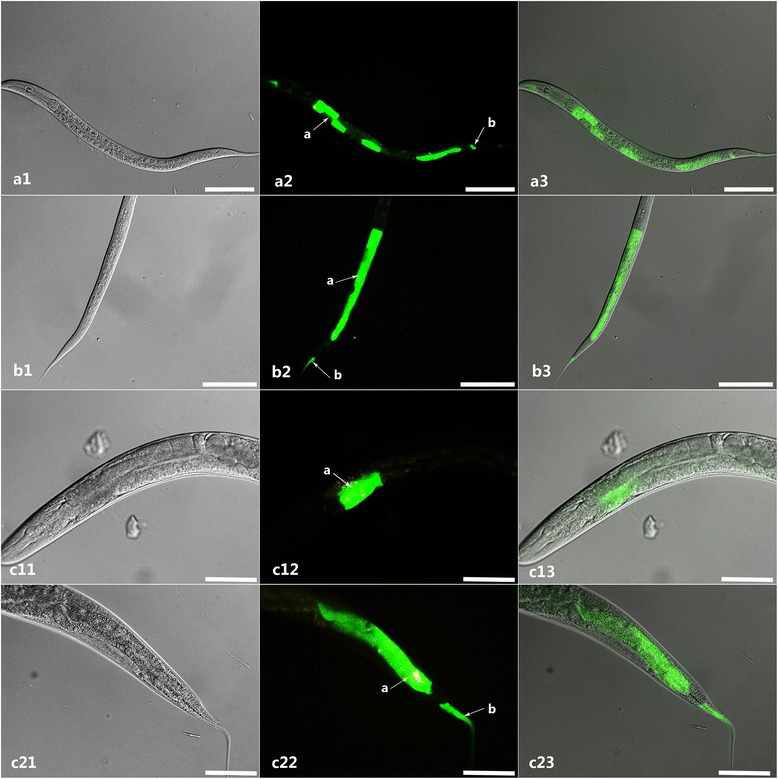
Expression of *Hc*-*daf*-*22* in *C. elegans daf*-*22* (*ok693*). The plasmid vector CeP-pPD95.77-*Hc*-*daf*-*22* was also injected into *C. elegans daf*-*22* (*ok693*). The GFP was located in the intestine (*arrow a*) and tail (*arrow b*). **a1**-**a3** L1 larvae. **b1**-**b3** L2 larvae. **c11**-**c23** Adult. *Scale*-*bars*: 200 μm

**Fig. 8:**
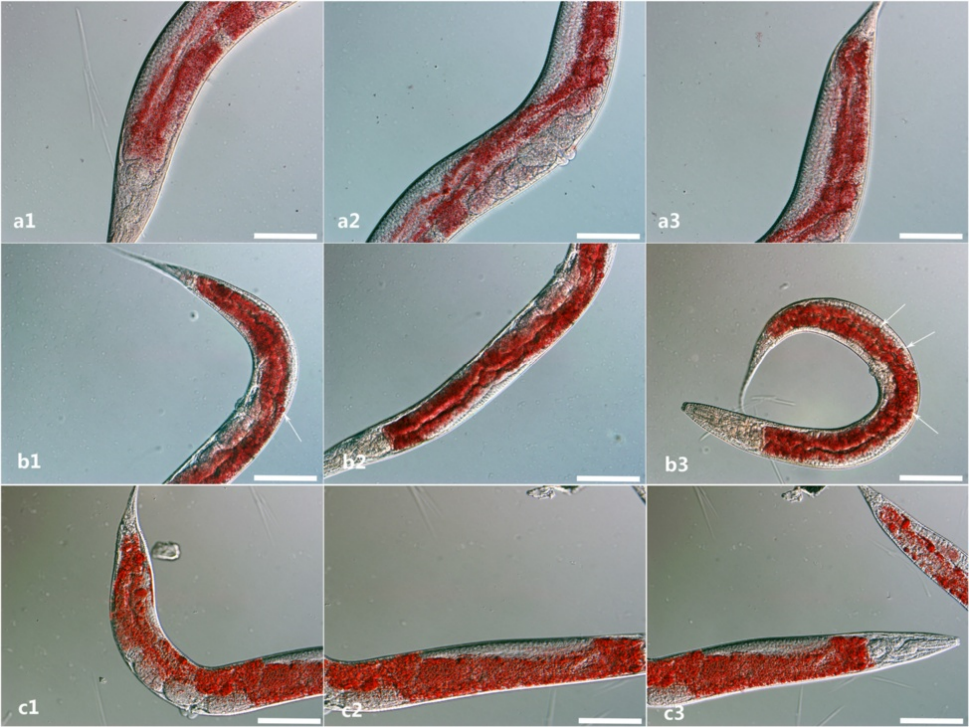
Fat storage and fat granule phenotype in *C. elegans* N2, *daf*-*22* (*ok693*), and rescued worms. **a1**-**a3** Fat storage in N2. **b1**-**b3** Fat storage and granules in *daf*-*22* (*ok693*) strain. **c1**-**c3** fat storage in rescued worms. Huge fat granules can be seen in the intestine of *daf*-*22* (*ok693*) (*arrows*), while fewer or even none seen in N2 and the rescued worm. Fat staining was performed with young adults of N2, *daf*-*22* (*ok693*), rescued worms using Oil red O. *Scale*-*bars*: 200 μm

**Fig. 9:**
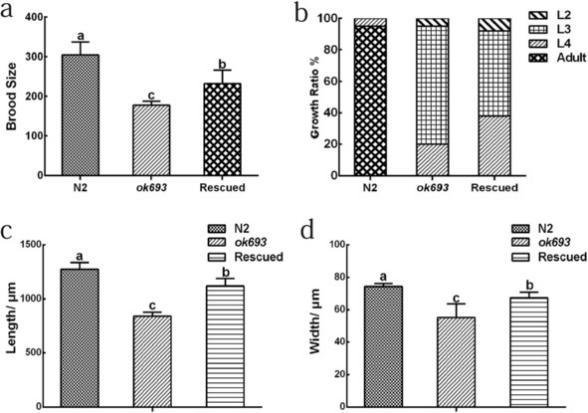
Post-embryonic developmental defects of *C. elegans* mutant was partially rescued by *Hc*-*daf*-*22*. **a** Brood size of N2, *daf*-*22* (*ok693*), rescued worms. *P*-values were calculated by Dunnett’s test. Significant differences are indicated with different letters (a, b, c) (*P* < 0.05). **b** Developmental growth ratio of N2, *daf*-*22* (*OK693*), rescued worms. Synchronized eggs were picked and the number of worms at each development stage was counted at 72 h. *P*-values were calculated by Dunnett’s test. **c** Body length of N2, *daf*-*22* (*ok693*), rescued worms. *P*-values were calculated by Dunnett’s test. Significant differences are indicated with (a, b, c) (*P* < 0.05). **d** Body width of N2, *daf*-*22* (*ok693*), rescued worms. *P*-values were calculated by Dunnett’s test. Significant differences are indicated with (a, b, c) (*P* < 0.05)

Based on existing data, *Ce*-*daf*-*22* mutant tends to accumulate enlarged spherical intracellular structures which were confirmed to be fat droplets as a distinct phenotype and to cause defect in post-embryonic development [22]. To testify whether *Hc*-*daf*-*22* could rescue the loss function of *Ce*-*daf*-*22* in *daf*-*22* (*ok693*) strain, *daf*-*22* (*ok693*) strain was transformed with *Hc*-*daf*-*22*::*gfp*. GFP could be detected in all life stages mainly in the intestine and tail parts of the worms (Fig. 7). The rescued transformed lines of the mutant strain exhibited reduced fat storage, especially significant reduction or total disappearance of huge fat granules (Fig. 8). In addition, there was a significant increase in the body length and width of young adults of the transformed lines compared to the *daf*-*22* (*ok693*) mutants (t-test: *t* = 10.79, *df* =17, *P* < 0.0001; *t* = 8.857, *df* = 17. *P* < 0.0001). However, these measurements remained significantly lower than those of the N2 strain (t-test: *t* = 5.023, *df* = 17. *P* < 0.0001; *t* = 5.550, *df* = 17. *P* < 0.0001) (Fig. 9c, d); the same pattern was observed for the brood size (t-test: *t* = 8.605, *df* = 8. *P* < 0.0001; *t* = 3.333, *df* = 7. *P* = 0.0125) (Fig. 9a). No significant difference was observed in the growth rate between *daf*-*22* (*ok693*) and rescued worms (t-test: *t* = 0.378, *df* = 4, *P* = 0.7247) and both were significantly lower in contrast with that of N2 strain (t-test: *t* = 166.3, *df* = 4. *P* < 0.0001; *t* = 144.5, *df* = 4; *P* < 0.0001). Furthermore, no worms could grow into adult stage either in *daf*-*22* (*ok693*) mutant or rescued worms (Fig. 9b).

### RNAi in *C. elegans* N2 strain

**Fig. 10:**
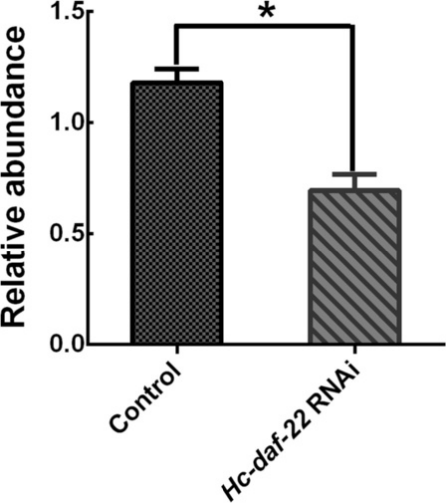
Change in mRNA level of RNA interference with L4440-*Hc*-*daf*-*22* in N2 *C. elegans*. The vector of L4440-*Hc*-*daf*-*22* was constructed and transformed into HT115 cells. Wild type *C. elegans* were grown on NGM plates seeded with the transformed bacteria, while the empty vector of L4440 was used as a negative control. Relative quantification of *Ce*-*daf*-*22* mRNA level of control N2 group and *Hc*-*daf*-*22* RNAi group (*P* < 0.05)

**Fig. 11:**
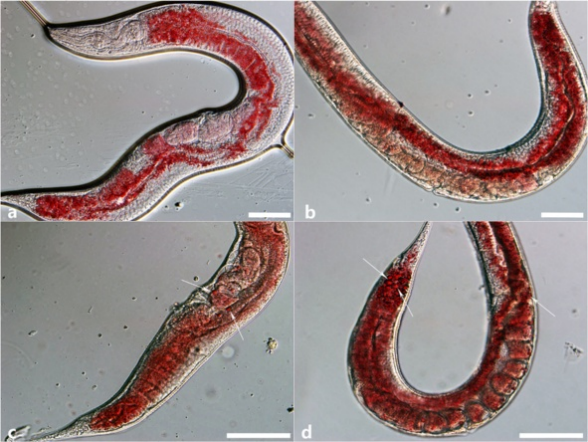
RNA interference with L4440-*Hc*-*daf*-*22* in N2 *C. elegans* by feeding. The adult worms were collected and stained with Oil red O. **a** Staining of N2 with no treatment. **b** Control adult feeding with empty L4440 stained with Oil red O. **c**, **d** Adults interfered by *Hc*-*daf*-*22* stained with Oil red O; huge fat granules are indicated by *arrows. Scale*-*bars*: 200 μm

RNAi experiment was carried out as described above to verify whether the *Hc*-*daf*-*22* gene could silence the endogenous mRNA of *Ce*-*daf*-*22* in *C. elegans* N2 strain. The relative quantification of mRNA levels of N2 and *Hc*-*daf*-*22* RNAi strain showed a significant reduction in gene transcription after RNAi with *Hc*-*daf*-*22* gene (t-test: *t* = 3.563, *df* = 4, *P* = 0.0235) (Fig. 10). Worms grown on *Hc*-*daf*-*22* (RNAi) plates (from egg to adult) at 20 °C were observed. There were no significant changes in body length, body width and growth rate compared with N2 worms (data not shown). However, the brood size of *Hc*-*daf*-*22* RNAi line was significantly decreased (t-test: *t* = 4.308, *df* = 4, *P* = 0.0126) and worms also displayed a phenotype that was richer in fat storage and accumulating more fat granules than the control worms (Fig. 11), as seen in the *daf*-*22* (*ok693*) mutants.

## Discussion

Recently, the whole genome of *H. contortus* was sequenced [23]; however, the precision and function annotation of the genes still need to be improved. In the present study, a new gene of *H. contortus*, *Hc*-*daf*-*22*, was isolated and characterized both in relation to gene structure and function. *Hc*-*daf*-*22* is composed by 16 exons separated by 15 introns, and this composition is identical to human [24] and mouse [25] SCPx genes. Comparison of amino acid sequences of Hc-DAF-22 among different species revealed that the thiolase domain and SCP2 domain were conserved in nematodes and mammals. The C-terminal of Hc-DAF-22 contained a tripeptide (Ala-Lys-Ile), which has already been proven to be functional in porcine species [26], in contrast to the peroxisomal-targeting signal 1 (PTS1; typically Ser-Lys-Leu [27, 28] that are conserved among yeasts, plants, insects and mammals. Moreover, the C-terminal of Hc-DAF-22 was also found containing the amino acids that are important for SCP2 sterol transfer function (Fig. 3). The SCPx domain in the N-terminal of Hc-DAF-22 was more close to *C. elegans* compared to other species; however, thiolase and SCP-2 domains in *C. elegans* are coded by two separate genes and not fused together as one protein [29]. These indicate that the SCPx domain is conserved through most species, while the fusion of SCP2 domain in the *Hc*-*daf*-*22* gene indicates a more advanced evolutionary position of the parasitic *H. contortus* in relation to the free-living *C. elegans*. Due to the similarities in structure and function with *Ce*-*daf*-*22* and other SCPx genes, *Hc*-*daf*-*22* may act as a peroxisomal protein and play an important role in worm development.

We obtained the 5′-flanking region of *Hc*-*daf*-*22* by genome walking to analyze sequence character and verify its promoter activity with the 5′-flanking region of *Ce*-*daf*-*22* as a control. Results from our analysis showed that the 5′-flanking region of *Hc*-*daf*-*22* contained a TATA box, GATA-1, CCAAT binding factor, CAC-binding protein, Unc-86, AP-1, AP-2, and NFγB sites, some of which can also be found in the promoter region of the SCPx gene in humans [30]. The micro-injection results indicated that this region shows promoter activity which could drive the GFP expression in the transformed lines in N2 strain mostly in the intestine area. However, compared to the control lines, the level of GFP expression was slightly low. Since the promoter region was forced to function in an alien environment of *C. elegans*, the driving ability could be affected and causing the relatively low GFP levels. In addition, the difference of gene structure may also lead to the difference between two promoters. *Hc*-*daf*-*22* contained two functional domains that are SCPx and SCP2 domains, while the *Ce*-*daf*-*22* only has one SCPx domain; therefore, it was reasonable to infer that *Hc*-*daf*-*22* function is more complicated than *Ce*-*daf*-*22* which may lead to a different driving pattern of the promoter. There are other possible explanations such as the first exon of *Hc*-*daf*-*22* has a distinct signal which may lead to different driving pattern of the promoter just like the first exon of human SCPx gene which has influence on the promoter region [31].

To demonstrate the gene function of *Hc*-*daf*-*22* in vivo, transformations of N2 and *daf*-*22* (*ok693*) strains with *cp*-*Hc*-*daf*-*22*::*gfp* were performed by micro-injection. The detection of GFP revealed that the recombinant Hc-DAF-22 could be expressed in the intestine region in a similar pattern to *Ce*-*daf*-*22* in *C. elegans* [18]. Besides, Hc-DAF-22 could partially restore the phenotype of the *daf*-*22 mutant* (*ok693*) as the fat granules were significantly reduced or even completely absent (Fig. 8). The subsequent analysis of the post-embryonic development showed that Hc-DAF-22 also partially rescued the body size and brood size of the *daf*-*22* (*ok693*) mutants (Fig. 9a, c, d), although no significant change was seen in growth rates. Similar results were observed by other researchers when expressing *Hc*-*hsp*-*90* in *C. elegans daf*-*21* mutant. Hc-HSP-90 could only partially rescue the phenotype of the mutant [31]. Nevertheless, other studies showed a full restore of transgenetic expression of parasitic genes in mutant strains of *C. elegans*. The expression of *A. caninum slo*-*1* and *C. oncophora slo*-*1* genes in *C. elegans slo*-*1*(*js379*) mutant was able to rescue the phenotype of worm locomotion and phenotypic behavior [32]. In another study, the glutamate-gated chloride channel (GluCl) subunit of *H. contortus* was expressed in a *C. elegans* mutant (*DA1316*), which was able to rescue the ivermectin sensitivity of mutant *C. elegans* [33]. These data indicate that the expression of parasitic genes in mutants of *C. elegans* could provide a first step in understanding gene functions in vivo, some genes could fully rescue the functions of *C. elegans* mutant while some could not. The similarity of parasitic genes with the homologues in *C. elegans* may play a key role in the successful rate of rescue. In our case, the partial rescue of *daf*-*22* (*ok693*) mutant with *Hc*-*daf*-*22* may be caused by the difference of gene structure between *Hc*-*daf*-*22* and *Ce*-*daf*-*22* as described above.

RNA interference (RNAi), inducing gene silencing, has been applied successfully to *C. elegans* and provides a functional genomic platform in a range of organisms including parasitic nematodes. Although RNAi experiments in parasitic nematodes such as in *Ostertagia ostertagi* [34], *Heligmosomoides polygyrus* [35], *H. contortus* [36] have been successful, there is still a limited number of genes which can be successfully silenced with RNAi due to many complicated reasons, including the lack of appropriate methods of dsRNA delivery and in vitro culture systems for parasitic nematodes, differences in RNAi effector protein functionality and in the complement of RNAi effectors between nematodes [33, 35, 37, 38]. In this study, we used RNAi to silence the *Ce*-*daf*-*22* of *C. elegans* with *Hc*-*daf*-*22* by feeding. Based on our observation, the *daf*-*22* (RNAi) phenotype showed more fat storage and larger fat granules in a manner similar to *daf*-*22* (*ok693*) mutant strain. Furthermore, the relative quantification of mRNA levels showed that *Hc*-*daf*-*22* could successfully partially silence the *Ce*-*daf*-*22*, which further confirmed the similarity of gene structure and possible functions between these two genes.

Based on the expression pattern in different stages of *H. contortus*, it was noteworthy that *Hc*-*daf*-*22* reached its peak in L3 and L4 stages. L3 stage worms have sealed mouth and live without feeding, so it is very important for them to take full advantage of the inner sources of energy like fat to survive, while L4 worms are just starting to feed on blood; this gene may play important roles in non-feeding stages of *H. contortus*.

In this study, we demonstrated that *Hc*-*daf*-*22* has similar gene structure with the SCPx protein genes, which all act as peroximal β-oxidation enzymes associated in the fatty acid metabolism. We also confirmed that *Hc*-*daf*-*22* could partially recued the function of *Ce*-*daf*-*22* in *daf*-*22* mutant (*ok693*). It is safe to infer that *Hc*-*daf*-*22* may play a similar role in the same pathway in *H. contortus*. Further studies are needed to confirm its enzyme activity as thiolase, its position in the peroximal β-oxidation pathway and its relationship with diapause formation in *H. contortus* in developmental level. The results of our study primary demonstrated the possible role of this gene and showed a promising future in the research of parasitic diapause formation mechanisms.

## Conclusion

In this study, a new gene *Hc*-*daf*-*22* was identified which is the homologue of *Ce*-*daf*-*22* and human SCPx. Genome walking and RACE confirmed the full length gene of *Hc*-*daf*-*22* (6,939 bp) which contained 16 exons separated by 15 introns, and encoded a cDNA of 1,602 bp. The 1,548 bp fragment upstream of the 5′-flanking region was confirmed to have promoter activity compared with 5′-flanking region of *Ce*-*daf*-*22* by micro-injection. The rescue experiment indicated that *Hc*-*daf*-*22* could partially rescue the function of *Ce*-*daf*-*22*. Furthermore, RNAi with *Hc*-*daf*-*22* could partially silence the endogenous *Ce*-*daf*-*22* in N2 worms and mimic the phenotype of *daf*-*22* (*ok693*) mutants. *Hc*-*daf*-*22* shared similar characteristics and function with *Ce*-*daf*-*22* and may play an important role in peroxisomal β-oxidation and development in *Haemonchus contortus*.

## Abbreviations

Not applicable.

## References

[1] Golden JW, Riddle DL (1984). The *Caenorhabditis elegans* dauer larva: developmental effects of pheromone, food, and temperature. Dev Biol.

[2] Sommerville RI, Davey KG (2002). Diapause in parasitic nematodes: a review. Can J Zoo.

[3] Butcher RA, Ragains JR, Li W, Ruvkun G, Clardy J, Mak HY (2009). Biosynthesis of the *Caenorhabditis elegans* dauer pheromone. Proc Natl Acad Sci U S A.

[4] Bun-ya M, Maebuchi M, Kamiryo T, Kurosawa T, Sato M, Tohma M (1998). Thiolase involved in bile acid formation. J Biochem-Tokyo.

[5] Barnes TM, Jin Y, Horvitz HR, Ruvkun G, Hekimi S (1996). The *Caenorhabditis elegans* behavioral gene *unc*-*24* encodes a novel bipartite protein similar to both erythrocyte band 7.2 (stomatin) and nonspecific lipid transfer protein. J Neurochem.

[6] Wirtz KWA (1991). Phospholipid transfer proteins. Annu R Biochem.

[7] Gallegos AM, Atshaves BP, Storey SM, Starodub O, Petrescu AD, Huang H (2001). Gene structure, intracellular localization, and functional roles of sterol carrier protein-2. Prog Lipid Res.

[8] Ossendorp BC, Heusden GPH, Beer ALJ, Bos K, Schouten GL, Wirtz KWA (1991). Identification of the cDNA clone which encodes the 58-kDa protein containing the amino acid sequence of rat liver non-specific lipid-transfer protein (sterol-carrier protein 2). Eur J Biochem.

[9] Mannaerts GP, Van Veldhoven PP, Casteels M (2000). Peroxisomal lipid degradation via β-and α-oxidation in mammals. Cell Biochem.

[10] Kitamura T, Kobayashi S, Okada M (1996). Regional expression of the transcript encoding sterol carrier protein x-related thiolase and its regulation by homeotic genes in the midgut of *Drosophila* embryos. Dev Growth Differ.

[11] Wanders RJA, Denis S, Wouters F, Wirtz KWA, Seedorf U (1997). Sterol carrier protein X (SCPx) is a peroxisomal branched-chain β-ketothiolase specifically reacting with 3-oxo-pristanoyl-CoA: a new, unique role for SCPx in branched-chain fatty acid metabolism in peroxisomes. Biochem Bioph Res Co.

[12] Wanders RJA, Denis S, Van Berkel E, Wouters F, Wirtz KWA, Seedorf U (1998). Identification of the newly discovered 58 kDa peroxisomal thiolase SCPx as the main thiolase involved in both pristanic acid and trihydroxycholestanoic acid oxidation: Implications for peroxisomal β-oxidation disorders. J Inherit Metab Dis.

[13] Ghedin E, Wang S, Spiro D, Caler E, Zhao Q, Crabtree J (2007). Draft genome of the filarial nematode parasite *Brugia malayi*. Science.

[14] Bairoch A (1993). The PROSITE dictionary of sites and patterns in proteins, its current status. Nucleic Acids Res.

[15] Bateman A, Coin L, Durbin R, Finn RD, Hollich V, Griffiths‐Jones S (2004). The Pfam protein families database. Nucleic Acids Res.

[16] Kumar S, Stecher G, Tamura K (2016). MEGA7: Molecular Evolutionary Genetics Analysis version 7.0 for bigger datasets. Mol Biol Evol.

[17] Mörck C, Pilon M (2006). *C. elegans* feeding defective mutants have shorter body lengths and increased autophagy. BMC Dev Biol.

[18] Joo H-J, Yim Y-H, Jeong P-Y, Jin Y-X, Lee J-E, Kim H (2009). *Caenorhabditis elegans* utilizes dauer pheromone biosynthesis to dispose of toxic peroxisomal fatty acids for cellular homoeostasis. Biochem J.

[19] Wong A, Boutis P, Hekimi S (1995). Mutations in the *clk*-*1* gene of *Caenorhabditis elegans* affect developmental and behavioral timing. Genetics.

[20] O’Rourke EJ, Soukas AA, Carr CE, Ruvkun G (2009). *C. elegans* major fats are stored in vesicles distinct from lysosome-related organelles. Cell Metab.

[21] Timmons L, Court DL, Fire A (2001). Ingestion of bacterially expressed dsRNAs can produce specific and potent genetic interference in *Caenorhabditis elegans*. Gene.

[22] Zhang SO, Box AC, Xu N, Le Men J, Yu J, Guo F (2010). Genetic and dietary regulation of lipid droplet expansion in *Caenorhabditis elegans*. Proc Natl Acad Sci U S A.

[23] Erich MS, Pasi KK, Bronwyn EC (2013). The genome and developmental transcriptome of the strongylid nematode *Haemonchus contortus*. Genome Biol.

[24] Ohba T, Rennert H, Pfeifer SM, He Z, Yamamoto R, Holt JA (1994). The structure of the human sterol carrier protein X/sterol carrier protein 2 gene (SCP2). Genomics.

[25] Seedorf U, Raabe M, Ellinghaus P, Kannenberg F, Fobker M, Engel T (1998). Defective peroxisomal catabolism of branched fatty acyl coenzyme A in mice lacking the sterol carrier protein-2/sterol carrier protein-x gene function. Genes Dev.

[26] Möller G, Lüders J, Markus M, Husen B, Van Veldhoven PP, Adamski J (1999). Peroxisome targeting of porcine 17β-hydroxysteroid dehydrogenase type IV/D-specific multifunctional protein 2 is mediated by its C-terminal tripeptide AKI. J Cell Biochem.

[27] Gould SJ, Keller G-A, Hosken N, Wilkinson J, Subramani S (1989). A conserved tripeptide sorts proteins to peroxisomes. J Cell Biol.

[28] Gould SJ, Keller G-A, Schneider M, Howell SH, Garrard LJ, Goodman JM (1990). Peroxisomal protein import is conserved between yeast, plants, insects and mammals. EMBO J.

[29] Pfeifer SM, Sakuragi N, Ryan A, Johnson AL, Deeley RG, Billheimer JT (1993). Chicken sterol carrier protein m2/sterol carrier protein x: cDNA cloning reveals evolutionary conservation of structure and regulated expression. Arch Biochem Biophys.

[30] Ohba T, Holt JA, Billheimer JT, Strauss Iii JF (1995). Human sterol carrier protein x/sterol carrier protein 2 gene has two promoters. Biochemistry.

[31] Gillan V, Maitland K, McCormack G, Him NAIIN, Devaney E (2009). Functional genomics of *hsp*-*90* in parasitic and free-living nematodes. Int J Parasitol.

[32] Welz C, Krüger N, Schniederjans M, Miltsch SM, Krücken J, Guest M (2011). SLO-1-channels of parasitic nematodes reconstitute locomotor behaviour and emodepside sensitivity in *Caenorhabditis elegans slo*-*1* loss of function mutants. PLoS Pathog.

[33] Glendinning SK, Buckingham SD, Sattelle DB, Wonnacott S, Wolstenholme AJ (2011). Glutamate-gated chloride channels of *Haemonchus contortus* restore drug sensitivity to ivermectin resistant *Caenorhabditis elegans*. PLoS One.

[34] Visser A, Geldhof P, De Maere V, Knox DP, Vercruysse J, Claerebout E (2006). Efficacy and specificity of RNA interference in larval life-stages of *Ostertagia ostertagi*. Parasitology.

[35] Lendner M, Doligalska M, Lucius R, Hartmann S (2008). Attempts to establish RNA interference in the parasitic nematode *Heligmosomoides polygyrus*. Mol Biochem Parasitol.

[36] Samarasinghe B, Knox DP, Britton C (2011). Factors affecting susceptibility to RNA interference in *Haemonchus contortus* and in vivo silencing of an H11 aminopeptidase gene. Int J Parasitol.

[37] Knox DP, Geldhof P, Visser A, Britton C (2007). RNA interference in parasitic nematodes of animals: a reality check?. Trends Parasitol.

[38] Viney ME, Thompson FJ (2008). Two hypotheses to explain why RNA interference does not work in animal parasitic nematodes. Int J Parasitol.

